# Editorial: Impact of dairy farming on the safety of raw milk and milk products

**DOI:** 10.3389/fmicb.2024.1491295

**Published:** 2024-09-20

**Authors:** Armin Tarrah, Lu Meng, Taurai Tasara, Tanushree B. Gupta

**Affiliations:** ^1^Dairy at Guelph, Canadian Institute for Food Safety, Department of Food Science, University of Guelph, Guelph, ON, Canada; ^2^Institute of Animal Sciences, Chinese Academy of Agricultural Sciences, Beijing, China; ^3^Institute for Food Safety and Hygiene, Vetsuisse Faculty, University of Zurich, Zurich, Switzerland; ^4^The Hopkirk Research Institute, AgResearch, Palmerston North, New Zealand

**Keywords:** milk safety, milk quality, microbial contamination, antimicrobial resistance, dairy

The dairy industry plays a crucial role in global food systems; however, ensuring the safety and quality of raw milk and milk products is a complex challenge. This Research Topic, “*Impact of dairy farming on the safety of raw milk and milk products*,” presents a collection of studies that explore various aspects of dairy safety. The papers featured delve into microbial contamination, antimicrobial resistance, and the relationship between environmental factors and milk microbiota. By examining these critical factors, this Research Topic aims to enhance our understanding of dairy safety and propose strategies for improving milk quality, ultimately contributing to better public health outcomes.

The study by Wilson et al. examines the microbial ecosystems in milk, tap water, and environmental swabs from a dairy farm and cheese processing facility over 6 months. The research highlights the impact of microbial contamination and biofilm accumulation in milking systems on the microbial quality of milk. The study found that while viable aerobic counts in milk generally remained low, occasional spikes were observed. Analysis revealed distinct microbial communities across different sample types, with *Brevibacterium* and *Yaniella* present in all samples. The research emphasizes the stochastic nature of microbiota composition between the farm and the processing facility, revealing that *Actinomycetota* are more likely to transfer from the farm to the facility, while *Pseudomonas* spp. may be enriched during transport and within the facility. Despite the movement between facilities, milk maintained stable viable plate counts, underscoring the benefits of using sole milk sources and highlighting the importance of proper storage and temperature control. The study also found higher levels of post-cleaning residual contamination in the on-farm milking system compared to the facility, with particular concern for microbial loads in milking hoses. Persistent microbes, including *Pseudomonas, Lactococcus, Staphylococcus*, and *Brevibacterium*, were identified on surfaces in the facility between cleaning cycles. Shared sequences of a limited number of genera, such as *Brevibacterium* and *Yaniella*, were observed from farm to facility, as well as between milk, water, and environmental swabs. These genera, associated with cheese ripening, point to important intra-genus diversity that warrants further investigation into their functionality and diversity within dairy systems.

The study by Gelalcha et al. assesses the prevalence of extended-spectrum beta-lactamase (ESBL)-producing *Enterobacteriaceae*, specifically *Escherichia coli* and *Klebsiella pneumoniae*, in bulk tank milk from East Tennessee dairy farms. The findings reveal prevalence of multidrug-resistant ESBL-*E. coli* and ESBL-*K. pneumoniae*, which possess resistance and virulence genes often associated with mobile genetic elements. Notably, half of the ESBL-*E. coli* isolates are high-risk clones with disease-causing potential and advantageous genetic traits. Whole-genome sequencing (WGS) identifies a diverse bacterial population with seven serotypes, six sequence types (STs), and eight core-genome sequence types (cgSTs). The discovery of a new allele type for the IncFIA (HI1) plasmid in a novel ST of *K. pneumoniae* further enhances the understanding of strain and plasmid diversity. These findings underscore the public health risks of consuming raw milk, which may harbor multidrug-resistant and virulent bacteria capable of causing infections or transferring resistance genes to clinically relevant strains.

The study by Liu et al. investigates the genomic epidemiology of *Staphylococcus aureus* isolates from raw milk in Jiangsu Province, China. Analyzing 117 isolates from 1,062 samples using whole-genome sequencing (WGS), the research identified major lineages such as CC1-ST1 and CC97-ST97 and highlighted the presence of high-risk methicillin-resistant *S. aureus* (MRSA) strains, including MRSA-ST59. The isolates exhibited high levels of enterotoxin genes, robust biofilm formation, and notable antibiotic resistance. These findings reveal significant public health risks from the potential transmission of *S. aureus* between livestock and humans, emphasizing the urgent need for enhanced surveillance and control measures in the dairy industry.

The study by Si et al. explores the relationship between rumen microbiota and milk fat content in Holstein dairy cows, focusing on how microbial communities influence milk fatty acid profiles. Over 2 weeks, milk and rumen fluid were collected from 16 cows [eight with high milk fat (HF) and eight with low milk fat (LF)]. The research identified a higher abundance of specific rumen bacteria in HF cows, such as *Prevotellaceae*_UCG-001 and *Ruminococcus*_1, which were positively correlated with elevated levels of certain fatty acids in milk. This study underscores how rumen microbiota, potentially influenced by dairy farming practices such as feed and silage management, affects milk fat composition.

The final study by Sun et al. examines the bacterial diversity and composition in raw camel milk from Xinjiang, China, using single-molecule real-time sequencing technology. The research identified significant seasonal and regional variations in bacterial composition, with *Epilithonimonas* being the most abundant genus. The study revealed that bacterial communities were strongly linked to metabolic pathways, particularly those related to fat, vitamins, and amino acids. Additionally, the presence of lactic acid bacteria (LAB) with antibacterial and anti-tumor properties was noted. The findings underscore concerns about contamination risks from psychrophilic and pathogenic bacteria and establish a crucial theoretical foundation for improving camel milk quality and safety.

This Research Topic provides a comprehensive examination of critical issues impacting dairy safety and quality. The studies reveal significant concerns related to microbial contamination, antibiotic resistance, and environmental influences on milk composition. Wilson et al. emphasize the need for effective cleaning and monitoring in dairy systems to manage microbial contamination. Gelalcha et al. highlight the pressing public health risks associated with multidrug-resistant *Enterobacteriaceae* in raw milk, advocating for enhanced surveillance. Liu et al. underscore the potential health risks from high-risk *S. aureus* strains in raw milk and stress the importance of improved control measures. Si et al. demonstrate how rumen microbiota affects milk fat content, suggesting that farming practices can influence milk quality. Sun et al. reveal regional and seasonal variations in camel milk's bacterial diversity, pointing to both contamination risks and opportunities for quality improvement.

Collectively, these studies underscore the need for continued research and robust intervention strategies to ensure the safety and quality of dairy products, ultimately supporting better public health outcomes.

